# The effects of lumiracoxib 100 mg once daily vs. ibuprofen 600 mg three times daily on the blood pressure profiles of hypertensive osteoarthritis patients taking different classes of antihypertensive agents

**DOI:** 10.1111/j.1742-1241.2010.02346.x

**Published:** 2010-05

**Authors:** T M MacDonald, D Richard, K Lheritier, G Krammer

**Affiliations:** 1 Hypertension Research Centre, Division of Medicine and Therapeutics, Ninewells HospitalDundee, UK; 2 Novartis Pharma AGBasel, Switzerland

## Abstract

**Aims:**

To examine whether the blood pressure (BP) profiles of lumiracoxib and high-dose ibuprofen differed in patients treated with different classes of antihypertensive medications.

**Methods:**

A 4-week, multicentre, randomised, double-blind study has compared the effects of lumiracoxib 100 mg once daily (od) (*n* = 394) and ibuprofen 600 mg three times daily (tid) (*n* = 393) on ambulatory BP in osteoarthritis (OA) patients with controlled hypertension. Here, we present subgroup analyses for patients receiving different antihypertensive classes. The primary outcome was a comparison of the change in 24-h mean systolic ambulatory BP (MSABP) from baseline to week 4. Patients receiving angiotensin receptor blockers (ARBs) or angiotensin-converting enzyme inhibitors (ACEIs) represented the largest subgroups receiving antihypertensive monotherapy.

**Results:**

For patients receiving an ARB monotherapy, the least squares mean (LSM) 24-h MSABP at week 4 fell with lumiracoxib 100 mg od and increased with ibuprofen 600 mg tid, creating an estimated treatment difference of 8.1 mmHg in favour of lumiracoxib (p < 0.001). For patients receiving an ACEI and a beta-blocker monotherapy, the estimated treatment difference was 8.2 mmHg (p < 0.001) and 5.8 mmHg (p = 0.002) in favour of lumiracoxib respectively. These treatment differences were greater than observed in the overall population (5.0 mmHg in favour of lumiracoxib). In patients receiving diuretics or calcium channel blockers, treatment differences in MSABP were smaller and not statistically significant, although they remained in favour of lumiracoxib.

**Conclusion:**

Lumiracoxib 100 mg od resulted in less destabilisation of BP than high-dose ibuprofen 600 mg tid, and this effect was the greatest in subgroups treated with drugs blocking the renin-angiotensin system.

**What's known**

- Non-steroidal anti-inflammatory drugs (NSAIDs) and COX-2 inhibitors are known to increase BP in patients receiving antihypertensive medication.
- Increases in BP can vary according to the individual NSAID or COX-2 inhibitors used.

**What's new**

- The lumiracoxib (COX-2 inhibitor), does not increase BP compared with high-dose ibuprofen (an NSAID) in patients with OA and well-controlled hypertension.
- Differences in BP between lumiracoxib and ibuprofen vary with the class of antihypertensive agent.
- Considering both antihypertensive and OA pain medications might help minimise destabilisation of BP.

## Introduction

In patients with previously stable hypertension, some non-selective non-steroidal anti-inflammatory drugs (NSAIDs) and selective cyclo-ooxygenase-2 (COX-2) inhibitors have been shown to increase systolic blood pressure and diastolic blood pressure (SBP and DBP) (1–4). However, these increases in BP can vary according to the individual NSAID used (2,5;) and even between COX-2 inhibitors (6). Previous studies have reported that NSAIDs (1,2,7,8;) and the COX-2 inhibitor rofecoxib (9) interact with BP control in patients treated with antihypertensive medications and patients treated with some classes of antihypertensive medications seem to be affected to a greater degree than others (4,8;).

Lumiracoxib (Prexige®, Novartis Pharma AG, Basel, Switzerland) is a novel selective COX-2 inhibitor that has been shown to have a lower 24-h mean systolic ambulatory BP (MSABP) profile (5 mmHg lower) than ibuprofen in an ambulatory BP monitoring (ABPM) study in osteoarthritis (OA) patients aged ≥ 50 years with controlled hypertension (10,11;). To examine whether the BP profiles of lumiracoxib and ibuprofen differed in patients treated with different classes of antihypertensive medications, a *post hoc* analysis of this ambulatory BP study was conducted.

## Patients and methods

### Study design

This was a *post hoc* analysis of a 4-week, multicentre, randomised, double-blind, double-dummy, parallel-group study conducted in 79 centres in nine countries (the USA, Austria, Belgium, Finland, France, Germany, Sweden, the UK, and Brazil). The original trial was an ABPM study in patients randomised to receive lumiracoxib 100 mg once daily (od) or high-dose ibuprofen 600 mg three times daily (tid). The study design and results have been published previously (10,11;). Ibuprofen 600 mg was chosen as the comparator as it is an approved prescription-strength drug for the treatment of OA in most of the European countries and a tid was considered to be the most commonly prescribed dosing frequency (12). For lumiracoxib, the prescription dose of 100 mg od has been shown to provide at least comparable efficacy to celecoxib 200 mg od (13,14;). Celecoxib 200 mg od in turn has been shown to have efficacy similar to ibuprofen 800 mg tid (15). Therefore, lumiracoxib 100 mg od can be considered as (at least) comparable in terms of efficacy, which would in turn be a conservative approach for comparison of safety as done in our study. Thus, differences in BP profile observed appear to be relevant.

Briefly, during a 1-week run-in phase, eligible patients had their current analgesic therapy washed out and replaced with paracetamol (acetaminophen) 1000 mg tid. At the end of the run-in period, patients underwent ABPM for 24 h. Patients who were at least 80% compliant with the run-in paracetamol regimen (paracetamol tablets were dispensed at the beginning of the washout phase and patients returned remaining tablets at the end of the washout phase for compliance to be assessed by pill counts) and whose ABPM data met the quality control criteria were then randomised in equal ratio to receive lumiracoxib 100 mg od or ibuprofen 600 mg tid orally for 4 weeks, and paracetamol treatment was stopped. Ambulatory BP monitors were fitted by experienced individuals and three to six correlation readings were performed to ensure that mean systolic and diastolic ambulatory BP was ±10/7 mmHg of the mean office sphygmomanometer BP readings. Quality control procedures were performed automatically by the ambulatory BP monitor using the correlation readings. During the 4-week treatment period, no changes were allowed to the patient’s usual antihypertensive treatment, and no paracetamol rescue medication, NSAID or other potentially BP-modifying treatments were permitted.

### Patients

Male and female outpatients (aged ≥ 50 years) were included if they had symptomatic primary OA of the hand, hip, knee or spine, pain in the target joint classified as mild, moderate or severe according to a 5-point categorical scale, and were expected to need NSAID treatment for at least 6 weeks. Patients were also required to have controlled hypertension (mean sitting SBP < 140 mmHg and mean sitting DBP < 90 mmHg, measured as the mean of three seated standard office sphygmomanometer readings taken at 1-min intervals after a 5-min rest), to have been taking the same regular fixed dosing regimen of antihypertensive medication(s) for ≥ 3 consecutive months prior to screening and who were not expected to adjust antihypertensive medication during the study.

### Assessments

Ambulatory BP was measured every 20 min using a ‘Spacelabs 90207 ABP Monitor’ (Spacelabs, Issaquah, WA, USA) during the 24-h at baseline (prior to randomisation) and after week 4 of treatment (study end). For the purpose of calibration of the device, three to six readings were taken in patients in a seated position. If the mean difference between three simultaneous ABP monitor and office mercury column systolic BP readings were outside ±10 mmHg and diastolic BP readings outside ±7 mmHg, the ABPM unit was reprogrammed and the calibration repeated.

The primary objective was the comparison of the change from baseline in 24-h MSABP between the lumiracoxib and ibuprofen treatment groups at week 4. Secondary end-points included change from baseline in 24-h mean diastolic ambulatory BP (MDABP); change from baseline in MSABP and MDABP during the daytime (> 06:00 to 22:00); change from baseline in MSABP and MDABP during the night-time (> 22:00 to 06:00); the percentage of patients with a clinically relevant increase in ambulatory BP (increase of ≥ 5 mmHg in 24-h MDABP and/or increase of ≥ 10 mmHg in 24-h MSABP); and the percentage of patients with uncontrolled hypertension (increase in 24-h mean ambulatory BP from < 130/80 mmHg at baseline to ≥ 130 mmHg and/or ≥ 80 mmHg after 4 weeks of treatment). The definition for uncontrolled hypertension as assessed by ambulatory BP was lower than the office cuff measurement used to screen patients as ABP measurements are typically lower than office cuff BP measurements.

### Statistics

Sample size calculations have been reported previously (10,11;). All BP evaluations were performed on the intention-to-treat (ITT) population, i.e. all randomised patients who received ≥ 1 dose of the study medication and successfully completed the postbaseline ABPM. The BP changes were adjusted for centre and baseline level of blood pressure (BP). The change from baseline in 24-h MSABP and MDABP, and daytime and night-time MSABP and MDABP were all analysed using an analysis of covariance (ANCOVA) with treatment as main effect and the appropriate baseline measure as a covariate. Additional analyses (summary statistics) were also carried out for the change from baseline at week 4 in 24 h MSABP in the following subgroup: gender (male/female); Age (≤ 64 years/65–74 years/≥ 75 years); race (White/ Caucasian, Black/African American, Hispanic, other). Significant effect modifiers were included in the model. Data are presented as least squares means (LSMs) and 95% confidence intervals (CIs). Multiple logistic regression models were used for clinically relevant increase in ABP and incidence of uncontrolled hypertension using baseline 24-h MSABP and baseline 24-h MDABP as covariates.

A *post hoc* analysis was conducted for patients receiving angiotensin receptor blockers (ARBs), angiotensin-converting enzyme inhibitors (ACEIs), beta-blockers, calcium channel blockers or diuretics as monotherapy, and also for patients receiving any treatment with these antihypertensive agents, including monotherapy or free and fixed-dose combinations.

## Results

A total of 787 patients were randomised in the ABPM study. All patients in the overall population were accounted for in the subsequent *post hoc* subgroup analyses of patients receiving ARBs, ACEIs, beta-blockers, calcium channel blockers or diuretics as monotherapy and patients receiving any treatment with these classes either as monotherapy or in free or fixed-dose combinations. The analyses of interest were those patients receiving monotherapy for each antihypertensive class. Our analyses focus on the use of ARBs or ACEIs as monotherapy because these populations had the greatest number of patients in the monotherapy subgroups and therefore provided the most robust data. Data on any use of these antihypertensive agents are provided only for the primary end-point.

In the ITT population, fewer patients received any antihypertensive monotherapy (lumiracoxib, *n* = 166; ibuprofen, *n* = 150) than combinations of antihypertensive agents (lumiracoxib, *n* = 227 and ibuprofen, *n* = 239). An ARB monotherapy was received by 57 and 48 patients randomised to lumiracoxib and ibuprofen, respectively. Angiotensin-converting enzyme inhibitors were the only antihypertensive medications in 42 patients receiving lumiracoxib and 40 patients taking ibuprofen. Details of patient disposition for patients receiving ARBs or ACEIs as monotherapy are presented in Table 1.

**Table 1:** Patient disposition for patients receiving angiotensin receptor blocker or angiotensin-converting enzyme inhibitor monotherapy

	ARB monotherapy		ACEI monotherapy	
	Lumiracoxib 100 mg od (*N* = 57)	Ibuprofen 600 mg tid (*N* = 48)	Lumiracoxib 100 mg od (*N* = 42)	Ibuprofen 600 mg tid (*N* = 40)
Completed, *n* (%)	53 (93.0)	47 (97.9)	39 (92.9)	38 (95.0)
**Discontinued, *n* (%)**	4 (7.0)	1 (2.1)	3 (7.1)	2 (5.0)
Death	0 (0.0)	0 (0.0)	0 (0.0)	0 (0.0)
Adverse event	2 (3.5)*	0 (0.0)	1 (2.4)†	0 (0.0)
Unsatisfactory therapeutic effect	1 (1.8)	0 (0.0)	1 (2.4)	0 (0.0)
Subject withdrew consent	0 (0.0)	0 (0.0)	0 (0.0)	2 (5.0)
Protocol violation	1 (1.8)	1 (2.1)	0 (0.0)	0 (0.0)
Administrative problems	0 (0.0)	0 (0.0)	1 (2.4)	0 (0.0)

* Upper abdominal pain (*n* = 1), retinal detachment (*n* = 1).

† Bells palsy (*n* = 1).

ACEI, angiotensin-converting enzyme inhibitor; ARB, angiotensin receptor blockers; od, once daily; tid, thrice daily.

Baseline demographic and background characteristics were similar between treatment groups for patients receiving an ARB or an ACEI as monotherapy (Table 2). In addition, baseline SBP and DBP were similar between treatment groups in patients receiving an ARB or an ACEI monotherapy.

**Table 2:** Baseline demographics and disease characteristics for patients receiving an angiotensin receptor blockers or an angiotensin-converting enzyme inhibitor monotherapy

	ARB monotherapy		ACEI monotherapy	
Parameter	Lumiracoxib 100 mg od (*N* = 57)	Ibuprofen 600 mg tid (*N* = 48)	Lumiracoxib 100 mg od (*N* = 42)	Ibuprofen600 mg tid (*N* = 40)
Age (years), mean ± SD	64.3 ± 8.4	64.6 ± 9.0	64.1 ± 7.6	61.0 ± 6.7
Range (min–max)	50–86	50–85	50–83	50–77
**Age group, *n* (%)**				
≤ 64 years	30 (52.6)	24 (50.0)	23 (54.8)	28 (70.0)
65–74 years	19 (33.3)	16 (33.3)	15 (35.7)	11 (27.5)
≥ 75 years	8 (14.0)	8 (16.7)	4 (9.5)	1 (2.5)
**Gender, *n* (%)**				
Female	41 (71.9)	35 (72.9)	22 (52.4)	28 (70.0)
Male	16 (28.1)	13 (27.1)	20 (47.6)	12 (30.0)
**Race, *n* (%)**				
Caucasians	56 (98.2)	45 (93.8)	38 (90.5)	39 (97.5)
Black/African Americans	1 (1.8)	2 (4.2)	1 (2.4)	1 (2.5)
Others	0 (0.0)	1 (2.1)	3 (7.1)	0 (0.0)
Duration of OA (years), mean ± SD	4.9 ± 5.3	6.7 ± 7.0	4.5 ± 6.0	5.0 ± 5.3
**OA pain, *n* (%)**				
Mild	5 (8.8)	6 (12.5)	9 (21.4)	10 (25.0)
Moderate	31 (54.4)	23 (47.9)	19 (45.2)	14 (35.0)
Severe	21 (36.8)	19 (39.6)	14 (33.3)	16 (40.0)
**Patient’s global assessment of disease activity, *n* (%)**				
Very good	0 (0.0)	1 (2.1)	0 (0.0)	4 (10.0)
Good	6 (10.5)	9 (18.8)	12 (28.6)	4 (10.0)
Fair	33 (57.9)	30 (62.5)	18 (42.9)	22 (55.0)
Poor	18 (31.6)	8 (16.7)	12 (28.6)	10 (25.0)
Very poor	0 (0.0)	0 (0.0)	0 (0.0)	0 (0.0)
**Physician’s global assessment of disease activity, *n* (%)**				
Very good	1 (1.8)	0 (0.0)	0 (0.0)	0 (0.0)
Good	2 (3.5)	6 (12.5)	8 (19.0)	12 (30.0)
Fair	41 (71.9)	31 (64.6)	26 (61.9)	20 (50.0)
Poor	13 (22.8)	11 (22.9)	8 (19.0)	8 (20.0)
Very poor	0 (0.0)	0 (0.0)	0 (0.0)	0 (0.0)
Duration of hypertension (years), mean ± SD	6.9 ± 6.6	6.2 ± 5.9	7.5 ± 7.8	6.3 ± 5.4
Sitting BP* (mmHg), mean ± SD				
Systolic	130.5 ± 7.2	129.1 ± 8.5	131.1 ± 6.5	129.7 ± 10.0
Diastolic	77.8 ± 7.0	77.7 ± 8.0	78.9 ± 6.0	77.8 ± 6.8
**24-h ambulatory BP (mmHg), mean ± SD**				
Systolic	127.2 ± 12.4	127.4 ± 12.1	131.1 ± 12.0	127.6 ± 12.2
Diastolic	75.6 ± 8.2	75.8 ± 7.7	77.4 ± 7.7	75.9 ± 7.9

* As measured by office cuff sphygmomanometer.

ACEI, angiotensin-converting enzyme inhibitor; ARB, angiotensin receptor blockers; BP, blood pressure; OA, osteoarthritis; od, once daily; SD, standard deviation; tid, thrice daily.

### Ambulatory blood pressure monitoring

**Figure 1:**
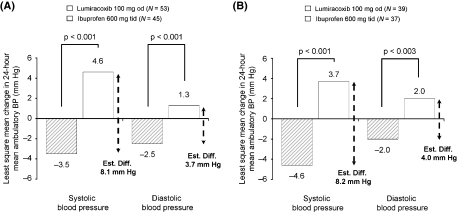
Change in 24-h mean systolic ambulatory blood pressure (mmHg) and mean diastolic ambulatory blood pressure (mmHg) from baseline with lumiracoxib and ibuprofen for 4 weeks in patients with well-controlled hypertension on (A) angiotensin receptor blocker monotherapy and (B) angiotensin-converting enzyme inhibitor monotherapy (ITT population). ACEI, angiotensin-converting enzyme inhibitor; ARB, angiotensin receptor blocker; Est. Diff., estimated treatment difference; ITT, intention-to-treat; MDABP, mean diastolic ambulatory blood pressure; MSABP, mean systolic ambulatory blood pressure; od, once daily; tid, three times daily. Treatment analysed using an analysis of covariance (ANCOVA) with treatment as main effect and centre and baseline 24-h MSABP as covariates

**Figure 2:**
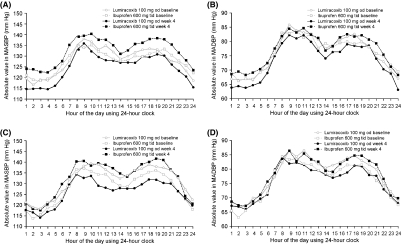
Change in mean systolic ambulatory blood pressure (A, C) and mean diastolic ambulatory blood pressure (B, D) with lumiracoxib and ibuprofen over the 24-h assessment periods (mmHg) at baseline and week 4 in patients with well-controlled hypertension on ARB monotherapy (A, B) or ACE monotherapy (C, D). ACEI, angiotensin-converting enzyme inhibitor; ARB, angiotensin receptor blocker; BP, blood pressure; MDABP, mean diastolic ambulatory blood pressure; MSABP, mean systolic ambulatory blood pressure; od, once daily; tid, thrice daily

Estimated treatment differences in 24-h MSABP for ARBs, ACEIs, beta-blockers, calcium channel blockers, diuretics and all antihypertensive agents as monotherapy, and for these antihypertensive classes including free and fixed-dose combinations, are listed in Table 3. For patients receiving an ARB monotherapy, the LSM change from baseline in 24-h MSABP after a 4-week treatment fell by 3.5 mmHg with lumiracoxib 100 mg od and increased by 4.6 mmHg with ibuprofen 600 mg tid, resulting in a statistically significant estimated treatment difference of 8.1 mmHg in favour of lumiracoxib (Figure 1A; Table 3). Figure 2 shows MSABP for lumiracoxib 100 mg od and ibuprofen 600 mg tid over the 24-h assessment periods at baseline and week 4 for patients receiving an ARB as monotherapy or ACEIs as monotherapy. For patients taking ACEI monotherapy, the LSM change from baseline in 24-h MSABP after 4 weeks of treatment fell by 4.6 mmHg with lumiracoxib 100 mg od and increased by 3.7 mmHg with ibuprofen 600 mg tid, resulting in a statistically significant estimated treatment difference of 8.2 mmHg in favour of lumiracoxib (Figure 1B; Table 3). These treatment differences in 24-h MSABP were greater than that observed in the overall ITT population (−2.7 mmHg decrease with lumiracoxib, a 2.2 mmHg increase with ibuprofen, producing an estimated treatment difference of 5 mmHg in favour of lumiracoxib). For patients receiving beta-blockers as monotherapy, there was a significant estimated treatment difference of 5.8 mmHg in 24-h MSABP in favour of lumiracoxib (Table 3). The use of diuretics or calcium channel blockers as monotherapy was associated with smaller, non-significant treatment differences in 24-h MSABP in favour of lumiracoxib (Table 3). For all antihypertensive monotherapy, there was an estimated treatment difference of 6.7 mmHg in favour of lumiracoxib for 24-h MSABP (Table 3). For any use of each antihypertensive class, including monotherapy and combinations, the 24-h MSABP at week 4 was significantly lower with lumiracoxib than with ibuprofen (Table 3). The estimated difference in 24-h MSABP between lumiracoxib and ibuprofen was 3.9 mmHg (in favour of lumiracoxib) for all patients receiving combination antihypertensive therapy (Table 3).

**Table 3:** Summary of the 24-h mean systolic ambulatory blood pressure (mmHg) assessments at week 4 with various antihypertensive treatments (ITT population)*

	LSM (SE) change from baseline at week 4					
Parameter	*N*	Lumiracoxib 100 mg od	*N*	Ibuprofen 600 mg tid	Estimated difference (95% CI)	p-Value
Overall population	363	−2.7 (0.4)	359	2.2 (0.4)	−5.0 (−6.1 to −3.8)	< 0.001
**Subgroups**						
**ARB**						
Monotherapy	53	−3.5 (1.2)	45	4.6 (1.3)	−8.1 (−11.5, −4.7)	< 0.001
Any use†	138	−2.6 (0.7)	130	3.5 (0.8)	−6.1 (−8.2, −4.0)	< 0.001
**ACEI**						
Monotherapy	39	−4.6 (1.3)	37	3.7 (1.4)	−8.2 (−12.1, −4.4)	< 0.001
Any use†	105	−4.0 (0.7)	110	2.0 (0.7)	−5.9 (−7.9, −3.9)	< 0.001
**Beta-blocker**						
Monotherapy	35	−3.0 (1.1)	22	2.8 (1.4)	−5.8 (−9.3, −2.3)	0.002
Any use†	109	−2.4 (0.7)	109	1.6 (0.7)	−4.0 (−6.0, −2.1)	< 0.001
**Calcium channel blockers**						
Monotherapy	17	−1.0 (1.4)	17	1.8 (1.4)	−2.8 (−6.9, 1.4)	0.184
Any use†	85	−2.3 (0.7)	66	1.2 (0.8)	−3.4 (−5.4, −1.5)	< 0.001
**Diuretic**						
Monotherapy	12	−1.5 (2.3)	17	2.1 (1.9)	−3.6 (−9.8, 2.6)	0.241
Any use†	183	−2.4 (0.6)	194	1.4 (0.6)	−3.8 (−5.4, −2.3)	< 0.001
**Any**						
Monotherapy	156	−3.2 (0.6)	138	3.4 (0.7)	−6.7 (−8.5, −4.9)	< 0.001
Combination‡	206	−2.5 (0.5)	217	1.3 (0.5)	−3.9 (−5.3, −2.4)	< 0.001

* Primary end-point.

† Monotherapy or free and fixed-dose combinations.

‡ Free or fixed-dose.

ACEI, angiotensin-converting enzyme inhibitor; ARBs, angiotensin receptor blockers; CI, confidence interval; MSABP, mean systolic ambulatory blood pressure; ITT, intention-to-treat; LSM, least squares mean; od, once daily; SE, standard error; tid, thrice daily.

Lumiracoxib 100 mg od had lower 24-h MDABP compared with ibuprofen 600 mg tid after the 4-week treatment resulting in a statistically significant estimated treatment difference of 3.7 mmHg in favour of lumiracoxib in patients taking ARBs only (−2.5 mmHg with lumiracoxib 100 mg od; +1.3 mmHg with ibuprofen 600 mg tid [p < 0.001]; Figure 1 and Table 3) and 4.0 mmHg in favour of lumiracoxib in patients receiving ACEIs only (−2.0 mmHg with lumiracoxib 100 mg od and +2.0 mmHg with ibuprofen 600 mg tid [p = 0.003]; Table 3). Lumiracoxib 100 mg od also demonstrated statistically significantly lower daytime and night-time MSABP and MDABP than ibuprofen in patients taking ARBs or ACEIs only (Table 4).

**Table 4:** Summary of the ambulatory BP measurement assessments (mmHg) for patients receiving angiotensin receptor blockers or an angiotensin-converting enzyme inhibitor monotherapy at week 4 (ITT population)

	LSM (SE) change from baseline at week 4					
Parameter	*N*	Lumiracoxib 100 mg od	*N*	Ibuprofen 600 mg tid	Estimated difference (95% CI)	p-Value
24-h MDABP*						
ARB monotherapy	53	−2.5 (0.6)	45	1.3 (0.7)	−3.7 (−5.6, −1.9)	< 0.001
ACEI monotherapy	39	−2.0 (0.9)	37	2.0 (0.9)	−4.0 (−6.6, −1.4)	0.003
Daytime MSABP*						
ARB monotherapy	53	−3.1 (1.3)	45	5.2 (1.4)	−8.3 (−12.2, −4.5)	< 0.001
ACEI monotherapy	39	−5.3 (1.6)	37	4.1 (1.6)	−9.4 (−13.9, −4.9)	< 0.001
Night-time MSABP*						
ARB monotherapy	53	−3.9 (1.2)	45	3.3 (1.3)	−7.2 (−10.6, −3.8)	< 0.001
ACEI monotherapy	39	−2.9 (1.39)	37	2.5 (1.42)	−5.4 (−9.4, −1.4)	0.008
Daytime MDABP*						
ARB monotherapy	53	−2.2 (0.8)	45	1.4 (0.8)	−3.6 (−5.8, −1.4)	0.001
ACEI monotherapy	39	−2.4 (1.1)	37	1.9 (1.1)	−4.3 (−7.4, −1.1)	0.009
Night-time MDABP*						
ARB monotherapy	53	−2.8 (0.7)	45	0.9 (0.7)	−3.7 (−5.6, −1.8)	< 0.001
ACEI monotherapy	39	−1.0 (1.0)	37	1.9 (1.0)	−2.8 (−5.7, 0.0)	0.049

* Change from baseline at week 4.

ACEI, angiotensin-converting enzyme inhibitor; ARB, angiotensin receptor blockers; BP, blood pressure; CI, confidence interval; ITT, intention-to-treat; LSM, least squares mean; MDABP, mean diastolic ambulatory blood pressure; MSABP, mean systolic ambulatory blood pressure; od, once daily; SE, standard error; tid, three times daily.

For patients receiving ARBs as monotherapy, the proportion of patients with a clinically relevant increase in BP was significantly greater for ibuprofen (33.3%) vs. lumiracoxib (5.7%) (odds ratio [OR] 0.12; 95% CI 0.0, 0.4; p = 0.002). The incidence of uncontrolled hypertension was smaller for lumiracoxib vs. ibuprofen (10.7% vs. 30.4%), but this difference was not statistically significant (OR 0.22; 95% CI 0.0, 1.2; p = 0.077).

The proportion of patients with a clinically relevant increase in BP was 21.6% for ibuprofen vs. 12.8% for lumiracoxib for patients receiving ACEIs as monotherapy, although the difference between treatment groups did not reach statistical significance (OR 0.63; 95% CI 0.2, 2.3; p = 0.486). The incidence of uncontrolled hypertension was smaller for lumiracoxib vs. ibuprofen (20.0% vs. 42.1%), but this difference was not statistically significant (OR 0.25; 95% CI 0.0, 1.6; p = 0.142).

In the overall study population, lumiracoxib 100 mg od and ibuprofen 600 mg tid treatment had comparable effects on measures of efficacy (pain intensity, patient’s global assessment of disease activity and physician’s global assessment of disease activity) (10). Similarly, in the ARB as monotherapy subgroup, there was no significant difference in efficacy between treatments (Table 5). For patients receiving ACEIs as monotherapy, the patient’s global assessment of disease activity and the physician’s global assessment of disease activity were not statistically different between lumiracoxib and ibuprofen. However, more patients had improvements in their OA pain intensity with lumiracoxib compared with ibuprofen (p = 0.032) in the ACEI monotherapy subgroup.

**Table 5:** Summary of patients improving efficacy variables on lumiracoxib and ibuprofen treatment at week 4 in subgroups of patients receiving angiotensin receptor blockers or an angiotensin-converting enzyme inhibitor monotherapy (ITT population)

	Patients improving score [*n*/*N* (%)]			
Parameter	Lumiracoxib 100 mg od	Ibuprofen 600 mg tid	Odds ratio (95% CI)	p-Value
**OA pain**				
ARB monotherapy	35/56 (62.5)	33/47 (70.2)	Not calculable*	0.531*
ACEI monotherapy	31/41 (75.6)	21/39 (53.8)	3.05 (1.1, 8.4)	0.032
**Patient’s global assessment of disease activity**				
ARB monotherapy	33/56 (58.9)	28/47 (59.6)	0.61 (0.2, 1.6)	0.298
ACEI monotherapy	25/41 (61.0)	18/39 (46.2)	Not calculable*	0.262*
**Physician’s global assessment of disease activity**				
ARB monotherapy	36/56 (64.3)	31/47 (66.0)	Not calculable*	> 0.999*
ACEI monotherapy	24/41 (58.5)	21/39 (53.8)	1.03 (0.3, 3.0)	0.955

In this analysis, treatment was the main effect and respective baseline variable was the covariate.

* If the model did not converge, the p-value was obtained using Fisher’s exact test.

ACEI, angiotensin-converting enzyme inhibitor; ARB, angiotensin receptor blockers; CI, confidence interval; ITT, intention-to-treat; od, once daily; tid, thrice daily.

## Discussion

This *post hoc* analysis suggests that lumiracoxib 100 mg od results in less destabilisation of SBP and DBP compared with high-dose ibuprofen 600 mg tid after the 4-week treatment in hypertensive OA patients aged ≥ 50 years, particularly when treated with an ARB or an ACEI monotherapy. Patients treated with an ARB or an ACEI represented the largest subgroups receiving antihypertensive monotherapy and the difference in LSM 24-h MSABP between lumiracoxib and ibuprofen was greater in these patients (ARB, −8.1 mmHg; ACEI, −8.2 mmHg) than for the overall population (−5.0 mmHg) (10). A significant difference in 24-h MSABP in favour of lumiracoxib was also observed in patients receiving beta-blocker monotherapy (−5.8 mmHg). For the calcium channel blockers and diuretic monotherapy subgroups, treatment differences were smaller than in the overall study population and in the ARB and ACEI monotherapy subgroups.

Previous studies have noted that NSAIDs can increase BP in patients receiving antihypertensive medication (1,2,7,8;). The effects of NSAIDs and COX-2 inhibitors on increasing BP would appear to differ by the class of antihypertensive medication. We used 600 mg of ibuprofen tid in the present study, which can be considered high-dose, although the over-the-counter dose is 400 mg tid and the maximum daily dose prescribed is 2.4 g daily in the UK.

In the present study, treatment with an ARB or an ACEI was associated with the greatest treatment differences in 24-h MSABP between lumiracoxib and ibuprofen. Other studies would indicate that the antihypertensive effect of ARBs and ACEIs is particularly susceptible to interference by NSAIDs (7,16;). The mechanism behind the interference of the antihypertensive effect of ARBs and ACEIs by NSAIDs is not clear from our study. However, given the similar magnitudes of the treatment differences with these antihypertensive classes and their mechanisms of action on the renin-angiotensin-aldosterone system, it seems probable that a common mediator, angiotensin II, is involved. Indeed, inhibition of prostaglandins has been reported to increase the sensitivity of renal blood vessels to angiotensin II (17). In healthy subjects, indomethacin has also been shown to abolish the natriuretic effect of an ARB or an ACEI therapy (18), and both these agents are equally susceptible to the deterioration in renal function with cyclo-oxygenase inhibition (19).The nature of the cyclo-oxygenase inhibitor is also an important factor, as evidenced by the treatment differences observed between lumiracoxib and ibuprofen in the current study. In the Successive Celecoxib Efficacy and Safety (SUCCESS)-VII study, rofecoxib was noted to increase BP more than celecoxib in patients treated with an ACEI or a beta-blocker, although there were no differences in BP between these COX-2 inhibitors in patients treated with a diuretic or a calcium channel blockers (4). The data reported from this study is consistent with the SUCCESS-VII study showing the greatest treatment differences with ARBs and ACEIs.

Antihypertensive monotherapy was associated with a greater difference in 24-h MSABP between lumiracoxib and ibuprofen than treatment with antihypertensive combinations (−6.7 mmHg vs. −3.9 mmHg). This is because the combinations tended to contain calcium antagonists or diuretics, drug classes that may be less affected by NSAIDs (20). This greater treatment difference with monotherapy also reflects the observation that ACEIs and ARBs comprised the majority of the monotherapy population.

The antihypertensive effect of calcium channel blockers appeared to be attenuated to a lesser extent by NSAID treatment. An ABPM study has shown that indomethacin increases BP more in patients treated with the ACE inhibitor, enalapril, than in patients receiving the calcium channel blockers, amlodipine (8). Another study has also reported that ibuprofen did not significantly increase BP in patients treated with the calcium channel blockers, verapamil (21). Numbers of patients in the calcium channel blocker subgroup of our study may have been too small to detect significant differences in ambulatory BP between lumiracoxib and ibuprofen in the present study.

Studies investigating the effects of NSAIDs on diuretic therapy would seem to indicate that the efficacy of these agents could be interfered with by NSAIDs. In a 4-week study, treatment with ibuprofen was reported to increase BP in patients treated with hydrochlorothiazide (22). Similarly, ibuprofen has been observed to increase SBP in elderly hypertensive patients on hydrochlorothiazide treatment (3).

As expected, given the difficulties of achieving BP control, more patients were receiving multiple antihypertensive therapies compared with monotherapy. Although diuretics represented the smallest subgroup for antihypertensive monotherapy in our study, they were the most commonly used agent in combinations and overall reflecting their status as a preferred and least expensive antihypertensive agent (23) with additive antihypertensive efficacy (24).

A recent meta analysis has indicated that lumiracoxib has a BP profile similar to placebo (25), suggesting that the difference in BP between lumiracoxib and ibuprofen in the current study is likely to be as a consequence of an adverse effect of ibuprofen on BP rather than an antihypertensive effect of lumiracoxib. Paracetamol treatment is known to increase BP (26) and therefore the fall in BP from baseline when paracetamol was replaced by lumiracoxib at the end of the 1-week run-in period would most likely indicate that lumiracoxib has a neutral BP profile. We cannot exclude that this BP fall was caused by prior withdrawal of NSAID therapy upon entry into the study or by withdrawal of paracetamol therapy given during the washout phase. It would have been good to have had a placebo treated arm in this study, but there were ethical issues surrounding the use of placebo in a group of patients requiring analgesia.

It remains unclear why there should be differences between lumiracoxib and high-dose ibuprofen on BP. Differences in pharmacokinetic and dosing regimen might be a possible explanation. For example, although both agents have a short half-life, the exposure of renal and cardiovascular tissues to ibuprofen might be greater as a result of the more frequent dosing regimen (tid). In contrast, lumiracoxib 100 mg was administered od, a regimen where it has previously been shown to be effective in treating OA pain (13,14;).

Given that the incidence of hypertension is greater in patients with OA than in the general population (27), minimising interactions between OA treatments and antihypertensive agent inhibitors might have benefits on the burden of healthcare. Indeed, avoiding significant increases in BP may have considerable long-term benefits for patients requiring chronic NSAID therapy for OA pain and should circumvent the need for additional BP monitoring and adjustments of antihypertensive medications. NSAIDs and selective COX-2 inhibitors have effects on systems other than BP, and therefore there is potential impact on toxicity in other organs. For example, it is known that lumiracoxib and other COX-2 inhibitors reduce the incidence of serious gastrointestinal ulcer complications compared with non-selective NSAIDs (28,29;).

A possible weakness of this analysis includes the fact that it was an exploratory and not a prespecified analysis of the original study. At the time of the randomization, there was no stratification according to these subgroups of antihypertensive agents; nevertheless, the distribution of therapies between treatments among subgroups was quite well distributed. Moreover, the numbers of patients receiving monotherapy with some classes of antihypertensive medications were small and could therefore confound drawing meaningful conclusions from this analysis. Finally, results presented in this manuscript are drawn from a 4-week study. This may not be long enough to draw definitive conclusions on the long-term cardiovascular outcome of treatment with lumiracoxib as compared with ibuprofen.

In conclusion, lumiracoxib 100 mg od resulted in less destabilisation of BP than high-dose ibuprofen 600 mg tid. This effect was maintained in most antihypertensive subgroups and was most pronounced in individuals receiving ARBs or ACEIs, the largest of the monotherapy subgroups.
